# Topical Application of Lidocaine and Bupivacaine to Disbudding Wounds in Dairy Calves: Safety, Toxicology and Wound Healing

**DOI:** 10.3390/ani11030869

**Published:** 2021-03-18

**Authors:** Meredith Sheil, Michael Chambers, Adam Polkinghorne, Brendan Sharpe

**Affiliations:** 1 Animal Ethics Pty. Ltd., Yarra Glen 3775, Australia; 2 Invetus Pty. Ltd., Armidale 2350, Australia; mchambers@invetus.com (M.C.); bsharpe@invetus.com (B.S.); 3 Department of Microbiology and Infectious Diseases, NSW Health Pathology, Nepean Blue Mountains Pathology Service, Penrith 2751, Australia; adam.polkinghorne@health.nsw.gov.au; 4 Nepean Clinical School, Faculty of Medicine and Health, University of Sydney, Kingswood 2747, Australia

**Keywords:** local anaesthetic, animal husbandry, thermocautery, wound infections, antiseptic

## Abstract

**Simple Summary:**

Disbudding is a common, but painful procedure performed on calves to prevent horn growth. Tri-Solfen^®^ is a combination local anaesthetic and antiseptic formulation which, applied topically to the disbudding wound, is reported to reduce calf pain. Applied in this manner, the local anaesthetics in Tri-Solfen^®^, lidocaine and bupivacaine, are reported to be poorly absorbed, resulting in low risk of neurological or cardiotoxic effects. The potential impacts on other blood, urine and tissue parameters and on wound healing when used in this manner, and/or accidental overdose situations are unknown, however. We performed experiments investigating (i) the safety of Tri-Solfen^®^ (including overdose situations) and (ii) the impact of Tri-Solfen^®^ on disbudding wound healing under field conditions. No adverse health effects were observed in Tri-Solfen^®^-treated animals, even those receiving 5× the recommended dose, with no clinically significant differences in measured parameters between placebo and Tri-Solfen^®^ groups. No negative impacts on wound healing were noted. Conversely, lower levels of bacterial wound colonisation were evident, and there was reduced incidence of abnormal wounds at days 11–12 in Tri-Solfen^®^-treated animals.

**Abstract:**

Tri-Solfen^®^ is a combination topical anaesthetic and antiseptic solution containing lidocaine, bupivacaine, adrenaline and cetrimide. Applied to wounds, it is reported to reduce the pain experienced by calves following thermocautery disbudding. While lidocaine and bupivacaine are widely used in medicine, conflicting data exist on the impact of these compounds when applied directly to the surgical wound. To investigate the safety of Tri-Solfen^®^ applied to thermocautery disbudding wounds of calves, experiments were performed to measure (i) the safety of Tri-Solfen^®^ (including in overdose situations); and (ii) the impact of Tri-Solfen^®^ application at recommended doses on disbudding wound healing under field conditions. Haematological, biochemical and urinalysis parameters did not show clinically significant differences between placebo and Tri-Solfen^®^ groups (1×, 3× and 5× dose). No adverse health impacts were reported. Histopathological analysis of wounds noted a reduction in bacterial colonies in Tri-Solfen^®^-treated wounds. Under field conditions, no negative impacts on wound healing were noted. Conversely, there was reduced incidence of abnormal wounds, with an associated trend toward improved average daily gain at days 11–12 in Tri-Solfen^®^-treated animals. These data are considered to support the safety of topical anaesthesia, as formulated in Tri-Solfen^®^, to the thermocautery disbudding wound in calves.

## 1. Introduction

Disbudding is an animal husbandry procedure conducted on-farm as part of routine management in cattle production enterprises [1]. Removal of the horn bud and horn-budding cells is considered to cause significant pain, based on a variety of outcome measures [2,3,4,5,6]. The use of topical anaesthesia, applied to the disbudding wound immediately post-procedure, is emerging as a simple and practical method to address post-operative pain. Tri-Solfen^®^, a combination topical anaesthetic and antiseptic formulation, is a licensed product in Australia and New Zealand, with a demonstrated ability to reduce post-operative hyperalgesic responses and pain-related behaviour in calves post-disbudding [7,8] and castration [9]. It contains local anaesthetics (50 g/L Lidocaine hydrochloride and 5 g/L Bupivacaine hydrochloride), a vasoconstrictor (0.048 g/L Adrenaline acid tartrate) and an antiseptic agent (5 g/L Cetrimide).

The veterinary medicines regulatory approval process requires, amongst other things, high standards of proof of safety of new animal medicines. Typically, this involves “Target Animal Safety”, as well as “field” safety studies, for which there are internationally harmonized (VICH) approved standards and guidelines [10]. The former is designed to examine detailed biochemical, haematological and histopathological impacts and also examine the impact of much higher dosages (e.g., 3–5× dosage) to understand the risk of potential misuse or accidental overdose. The latter are larger scale trials designed to examine safety of use in the field. The data generated from such trials can fill information gaps and be of high public value. This is particularly the case in terms of Tri-Solfen^®^ application to open wounds, such as the disbudding wound in calves. Although the local anaesthetics in Tri-Solfen^®^ have been widely used in human and veterinary medicine for many decades, there remains a dearth of information of the safety impacts, both local and systemic, when used in calves, and when applied topically to significant open wounds.

The acute systemic toxic effects of lidocaine and bupivacaine have been well described in humans and a variety of species (e.g., dogs, goat kids, lambs, pigs, horses and monkeys) [11]. Lidocaine and bupivacaine are amide local anaesthetics that alter neural signal conduction by blocking the fast voltage-gated Na+ channels in the neuronal cell membrane responsible for action potential propagation. Acute systemic toxic effects most commonly occur in the setting of rapid intravascular absorption and primarily manifest in the central nervous system (CNS) and/or cardiovascular system (CVS) at higher doses. Although the risk of acute systemic toxicity following topical application to intact skin is low due to poor penetration and low absorption [12], greater absorption and toxicity risk may occur if the agent is applied to mucus membranes or open wounds [13,14]. In view of this risk, until recently, topical local anaesthetics have been infrequently employed on significant open wounds, resulting in a lack of available safety data following use in this setting. Furthermore, in view of the fact that both lidocaine and bupivacaine were introduced well before today’s standards of safety testing, there is a paucity of available data on potential impacts on haematological and biochemical parameters and/or other organ systems. To begin to address these data gaps, we recently investigated the pharmacokinetics of topical administration of lidocaine and bupivacaine with adrenaline (as contained in Tri-Solfen^®^), to calf disbudding wounds immediately following thermocautery [15]. This study found that these local anaesthetics are poorly absorbed through the disbudding wound with peak plasma levels remaining well below toxic thresholds and declining quickly by 48 h post-administration. No clinical signs of CNS or CVS involvement were observed in any animals throughout the investigation. These results indicate very low risk of local anaesthetic systemic toxicity following topical application of Tri-Solfen^®^ to calves following thermocautery disbudding at the recommended dosage levels. Data on wider potential toxic effects in calves, or potential toxicity at higher levels of dosing remain lacking.

Questions also remain over potential local toxic impacts at the application site when topical local anaesthetics are applied to open wounds. Both anaesthetics may induce cytotoxic effects if delivered in high concentrations to sensitive tissues (e.g., cornea and cartilage [16]) which may have ramifications for their application to wounds and the potential impacts on wound healing. In terms of wounds, in vitro studies have shown that both local anaesthetics prevent cell growth and cause cell death at dilutions used in commercially available preparations [17,18]. In vivo data are more conflicting however, with some studies showing treatment with local anaesthetics may slow or prevent healing [19,20,21,22], while others report no effect [22,23,24,25,26], or, indeed, show a beneficial effect [27,28,29]. It is pertinent to note that detrimental effects on wound healing generally have only been reported following injected rather than topical anaesthetic application. Injections may disrupt tissue planes and deliver local anaesthetics into tissues under pressure, resulting in mechanical trauma that does not occur with topical application.

Furthermore, in vivo, any deleterious effects of local anaesthetics applied to wounds secondary to cytotoxicity have the potential to be counterbalanced by beneficial effects such as their potential anti-inflammatory and/or antimicrobial properties. Both bupivacaine and lidocaine are reported to have antimicrobial and inflammatory activity at therapeutic doses [30]. Accumulating data suggest that local anaesthetics may possess a wide range of anti-inflammatory actions through their “stabilising” effects on cells of the immune system, as well as on other cells (e.g., microorganisms, thrombocytes and erythrocytes) [31]. Although the detailed mechanisms of action are not fully understood, they have proved to be very successful in the treatment of burn injuries [31,32,33,34], and are reported to reduce risk of thrombo-embolism post-surgery [35]. Interestingly, in vitro studies have revealed that local anaesthetics in commercially available solutions also have bacteriostatic and/or bactericidal activity against equine bacterial pathogens [36]. In vivo studies, however, cast some doubt on the clinical effectiveness of lidocaine and bupivacaine as antimicrobial agents. No significant differences were observed in the inflammatory response or viable bacterial counts of rabbits experimentally inoculated with either *Escherichia coli* or *Staphylococcus aureus* and treated with either 0.9% NaCl, bupivacaine or lidocaine [37].

Tri-Solfen^®^ contains an antiseptic as well as the local anaesthetic actives. The impact of Tri-Solfen^®^ on wound healing has been reported in a range of wound types in different species. Lomax et al. reported an improved rate of healing over the first 14 days in lambs following tail docking and the flystrike prevention procedure of mulesing [38]. Sutherland et al. [39] reported that piglet castration wounds treated with Tri-Solfen^®^ healed as well as those treated with antiseptic alone, and better than those treated with a different local anaesthetic, without antiseptic. Most recently, Tri-Solfen^®^ treatment of disbudding wounds in calves suggested that its use may enhance wound healing compared to the alternative antimicrobial spray [40]. It is estimated that Tri-Solfen^®^ has now been used on >100 million animals, without reports of a negative impact on wound healing, including in a range of studies reporting its use in sheep [28,38], cattle [9] and pigs [41,42]; however, histopathological findings have not been reported.

In the current study, we report the results of studies examining (a) the target animal safety and (b) field safety of the application of Tri-Solfen^®^ to calf disbudding wounds including its impacts on wound healing and/or bacterial colonisation and infections.

## 2. Materials and Methods

### 2.1. Study Design

Assessment of the safety, toxicity and wound healing impacts of Tri-Solfen^®^ (50 g/L Lignocaine hydrochloride and 5 g/L Bupivacaine hydrochloride, 0.048 g/L Adrenaline acid tartrate, 5 g/L Cetrimide) administered to disbudding wounds in calves were evaluated in two major experiments. Study 1 was a blinded, randomised, controlled, parallel group “Target Animal Safety” (VICH-GL43) study. The study was designed to investigate the safety of Tri-Solfen^®^ applied to calf disbudding wounds, including at doses up to 5× the recommended dose. The study was designed in accordance with Good Laboratory Practice (GLP) [43,44,45,46] and target animal safety study [10] guidance documents. Ethics approval was provided by the University of New England Animal Ethics Committee, Armidale, Australia (19-078).

Study 2 was a blinded, randomised, controlled group field safety and efficacy trial. This study was designed to investigate the safety, including wound healing impacts, of Tri-Solfen^®^ applied at the upper recommended dose to calf disbudding wounds under field conditions. The study complied with the following national and international standards: (i) VICH GL9—Good Clinical Practice; (ii) APVMA Data Guidelines—Efficacy and target animal safety general guidelines. Approval for this project was provided by the University of New England Animal Ethics Committee, Armidale, Australia (19-095).

With regard to animal welfare, it is noted that calf disbudding is practiced under a range of different analgesic options in different jurisdictions globally. Although recommended, the use of appropriate anaesthetic and analgesic protocols is generally not compulsory. In a large proportion of cases globally, it is currently performed by farmers without any analgesia whatsoever [1]. Contributing to this state of affairs, there is a complete lack of medicines registered as safe and effective for pain alleviation in this setting in many jurisdictions. Internationally harmonised veterinary medicine regulatory requirements for the development of such medicines require studies of the stand-alone safety impacts of the medications [10]. The combined use of sedation, injected local anaesthesia, or systemic analgesia may confound systemic and/or local toxicity findings due to the topical anaesthetics alone, and therefore, are not employed in this setting.

### 2.2. Study 1—Target Animal Safety Study—Examining Safety of Tri-Solfen^®^ Administration to Calf Disbudding Wounds at up to 5× the Recommended Dose

The animal phase for Study 1 was completed between September and October 2019 at the University of New England Animal House, Armidale, Australia. Thirty-two (32) 2–7-weeks-old calves consisting of sixteen entire male and sixteen female animals from a population of approximately 50 mixed-sex dairy crossbred calves with confirmed hornbuds were selected for this study. The animals were stratified by sex and ranked from heaviest to lightest on bodyweight (recorded on day −4). They were then sequentially blocked into fours and randomly allocated to four treatment groups (placebo, 1×, 3×, 5× Tri-Solfen^®^ treatment) from within each block via random number draw.

The treatment regime for animals in Study 1 is outlined in Table 1. All calves were disbudded using a hot iron (18 mm, Kerbl Electric, Shoof International Pty Ltd., Cambridge, New Zealand). Following animal restraint, the heated hot iron was applied over the horn bud and rolled around the horn bud in a circular fashion several times, such that a ring of tissue around the bud was cauterised through the full thickness of the skin. Cauterised horn bud tissue (~20 mm diameter associated with each bud) was removed with forceps, providing a maximum absorptive bed of wounded tissue. Calves were treated following disbudding, allowing for 30 sec for cauterised tissue to cool. The three Tri-Solfen^®^ treatment groups then received dosages at 1×, 3× or 5× the maximum recommended dose of 2 mL per disbudding wound. To avoid large doses resulting in excessive run-off of the topical anaesthetic solution, 3× and 5× dosages were administered in repeated 2 mL aliquots approximately 1 hr apart to each disbudding wound. All doses were delivered within the 1–6 h T_max_ time frame reported for plasma and tissues of disbudded calves following lidocaine and bupivacaine treatments [15]. Additionally, all groups were re-treated on days 1 and 2 approximately 24 h and 48 h following their initial treatment, thus meeting GL43 guidelines requiring consideration of an extended duration of exposure [10]. Each disbudding wound in placebo animals was treated with 2 mL of a 0.9% sterile saline solution containing 1% blue food colouring to facilitate blinding.

From day −4, calves were housed in the same barn in individual adjacent pens each with wooden slat flooring. Calves n each treatment group were penned adjacent to one another with an empty pen between each treatment group. Calves’ diets were as per Dairy Australia accepted best practice. The level of roughage on offer followed current accepted best practice (https://www.dairyaustralia.com.au/en/animal-management-and-milk-quality/calf-rearing; accessed on 18 March 2021). Calves had ad libitum access to potable water. Half of the animals in each group were euthanised on day 3, with the remainder euthanised on day 4.

#### 2.2.1. Animal Observations Including Wound Assessment

Study animals were observed for the first 30 min following each treatment and thence hourly (±15 min) for a minimum of 6 h following the final treatment of each group on each day (0, 1 and 2). Twice daily observations, undertaken by a blinded veterinarian during the morning, continued prior to euthanasia on days 3 or 4. Observations included: general behaviour and demeanour, evaluation of any appetite change, ambulation, faecal consistency and colour, skin condition, ocular and nasal discharge, and any neurologic or cardiorespiratory signs that may be indicative of an adverse drug reaction. Bodyweights of animals were measured at day −4, and prior to euthanasia. Daily feed and water intake were also assessed on an individual animal basis once daily.

During each veterinary examination, disbudding wounds were examined for the presence of oedema, erythema, discharge, alopecia and flaking of skin on the area surrounding the cauterised wound. In addition, all application sites were photographed on the day of necropsy.

#### 2.2.2. Blood Sampling and Analysis

Blood specimens were collected from the jugular vein of carefully restrained calves at the following time points: day −4, day 0, and prior to euthanasia. Blood samples were collected by experienced veterinarians and/or technicians during the morning.

Blood was collected for coagulation, biochemical and haematological analysis. Serum and plasma were extracted after centrifugation (ROTOFIX 46, Hettich Asia Pacific Pty. Ltd, Singapore) at 3500 × rpm for 7 min at ambient temperature and stored in individual 5 mL vials. A thin blood smear was also prepared. Specimens (serum, plasma, whole blood in EDTA tubes) were then stored chilled and blood smears were stored at room temperature and forwarded to the laboratory (Gribbles Veterinary, Christchurch, New Zealand) for analysis. Standard biochemical, haematological and coagulation parameters were subsequently assessed in blood samples collected at day −4, 0 and the day of necropsy and compared to published reference ranges [47,48,49,50].

#### 2.2.3. Gross Pathology and Histopathology

Necropsy was performed on all animals by blinded and experienced veterinarians. Following visual examination, representative sections of all tissues (e.g., heart, brain, lung, liver, kidney, adrenal gland, application site tissue from the edge of the disbudding wound) were collected into 10% formal saline solution and stored for subsequent histopathological examination.

Sections of tissues underwent hematoxylin and eosin staining. Representative sections of all tissues were then examined from animals in Groups 1–4. Additionally, histopathology was evaluated for any organ with a gross abnormality from any animal. The complete list of tissues included application site skin, pituitary gland, thyroid gland, parathyroid gland, adrenal gland, pancreas, spleen, ovaries, uterus, testes, prostate, epididymis, heart, brain, brain stem, spinal cord, eye, lung, muscle, mammary gland, liver, gall bladder, kidney, urinary bladder, lymph node, skin, bone and marrow, marrow smear, stomach, duodenum, jejunum, ileum, colon, caecum and thymus. Evidence of pathology and/or the presence of multi-focal surface bacterial colonisation was scored as follows: 0 = normal, 1 = minimal, 2 = mild, 3 = moderate, 4 = marked.

#### 2.2.4. Urinalysis

Urine specimens (up to ~5 mL) were collected and recorded from all animals at necropsy on days 3 and 4 using a sterile needle and syringe directly from the bladder at autopsy. Detailed laboratory urinalysis was performed assessing a range of parameters including bilirubin, colour and clarity, glucose, ketones, pH, protein, specific gravity, urobilinogen, presence of blood (erythrocytes and haemoglobin) and microscopic examination of sediment.

#### 2.2.5. Statistical Analyses

Key clinical parameters, including heart rate, respiration rate, rectal temperature, bodyweight and bodyweight change, as well as key biochemical, haematological, urinary and coagulation parameters, were compared using SAS version 9.4 (SAS Institute, Cary, North Carolina, USA). Comparisons between groups were performed using covariance pattern repeated measures models and non-parametric tests, with treatment and time point and other variables assessed. Data from days 3 and 4 in Study 1 were combined into a single timepoint (“Sacrifice Day”).

Where available, pre-treatment data were included as a co-variate. Baseline data were defined as the latest measurement of a given variable before treatment was administered. The statistical model included treatment, sex (except for parameters only measured in one sex), and treatment-by-sex interactions as fixed effects, while the repeated measures ANOVA model included treatment, sex and time (and their 2-way and 3-way interactions) as fixed effects. Data were compared either within or across sex depending on observed interactions.

Models were selected using backwards elimination where 3-way and 2-way interaction terms with the highest *p*-Value were sequentially dropped from the model. The process was performed hierarchically starting with the 3-way interaction terms using a threshold *p*-Value of 0.1, as required by regulatory guidelines. Model selection was stopped when any of the following occurred: (i) all interaction terms at the highest level had a *p*-Value < 0.1; (ii) no interaction terms remained in the model. The statistical model was therefore:

Parameter = Baseline_variables + Treatment + Sex + Time + Treatment:Sex + Treatment:Time + Sex:Time + Treatment:Sex:Time + Residual_Error

Least squares means were compared at a significance level of *p* < 0.1 in the first instance apart from treatment by sex by time point, which was compared at *p* < 0.05 in the first instance.

For the repeated measures models, most residual error patterns were selected based on a comparison of Akaike Information Criteria (AIC) output for models containing an unstructured (UN), autoregressive (AR; 1), variance components (VC) and independent residual error pattern (with the lowest AIC preferred among the possible covariance matrices).

The denominator degrees of freedom in the covariance pattern repeated measures models were calculated using the Satterthwaite approximation. Model suitability was assessed by examination of residual plots, preliminary data exploration for normality or non-normality, tests for normality and results from homogeneity of variance tests. Data were not transformed with the exception of Creatine Kinase which was log-transformed.

Categorical urinalysis data were compared (proportions of animals/group in each category) using Fisher’s Exact Test across sex. Urinalysis data reported on a numerical scale (pH and specific gravity) were compared by Kruskal–Wallis test with exact *p*-Values estimated using Monte Carlo estimation.

### 2.3. Study 2—Safety and Efficacy of Tri-Solfen^®^ Administration to Calf Disbudding Wounds under Field Conditions

Animal experiments for Study 2 were completed between November and December 2019 on commercial dairy farms in Gloucester, New South Wales, Australia. Seventy-four (74) young female Holstein-Friesian and Jersey calves 2–6 weeks of age were selected from two similar dairy herds located within 20 km of each other. Calves were identified as suitable for selection based on confirmed overall good health and presence of normal, healthy horn buds. Animals were weighed, stratified by weight and herd of origin and randomly allocated into two treatment groups—placebo (*n* = 36) and Tri-Solfen^®^ (*n* = 38)—using “draw from a hat” methodology. Prior to disbudding and treatment, all calves were examined for safety assessments and sham-treated. Sham treatment involved restraining and handling as per disbudding but without actual disbudding or treatment and subsequent algometer (Force Cell Module 50 × 0.02 lbf; Wagner Instruments, Greenwich, CT, USA) and pain scoring on day −2 and day −1.

All calves were disbudded using a hot iron as described in Study 1. Following disbudding, a period of approximately 30 sec was allowed for the tissue to cool before application of the placebo (1 × 2 mL 0.9% saline solution with blue food dye) or Tri-Solfen^®^ (1 × 2 mL Tri-Solfen^®^) solution.

Trial calves were housed in covered pens (multiple calves/pen) for the duration of the study. Calves were provided with ad libitum access to potable water, pellets and roughage as per best practice at the trial sites.

Study animals were retained by their herd of origin at the conclusion of the in-life phase of the study at day 33–34.

#### 2.3.1. Clinical Examination

Detailed individual clinical examinations were performed on selected calves on day −2 and day −1 following sham-treatment. Clinical examinations were then repeated immediately prior to pain assessments at 22 h, days 7–8 and days 11–12. Examined parameters included gait and activity, eating/drinking, urination/defecation and general calf demeanour. Disbudding wounds were visually examined on days 3–4, days 7–8, days 21–22 and days 33–34. Disbudding wounds were assessed for overall appearance, wound resolution and the presence/absence of infection or other adverse clinical findings. Wounds were classified as either “normal” or “abnormal”. A classification of a normal wound was defined as the presence of a dry wound with epithelial contraction. An abnormal wound was defined as a wound with the presence of (i) an open wound; and/or (ii) necrotic tissue; and/or (iii) the presence of significant pus.

Calves were also weighed on days −2, days 11–12, days 21–22 and days 33–34 using electronic stock scales (Ruddweigh 300, Gallagher, Australia). Average daily gain was calculated on an individual animal basis over the periods pre-procedure to days 11–12, days 21–22 and days 33–34.

Efficacy measurements were initially planned as a part of Study 2, including wound sensitivity testing using a hand-held algometer (Force Cell Module 50 × 0.02 lbf; Wagner Instruments) and video recording of pain-related behaviour including ear flicks and head shakes. Although initially recorded, analysis was not progressed as extreme environmental conditions, including waves of fly infestation occurred, and were felt to have likely confounded and compromised the sensitivity and specificity of the proposed efficacy measurements.

#### 2.3.2. Statistical Analyses

Safety assessment parameter data (bodyweights, disbudding wound data and clinical examination data) were entered into Microsoft Excel (Microsoft, Redmond, WA, USA). All statistical comparisons were performed using Statistix 10.0 (Analytical Software). Average daily gain was calculated using individual animal weights at the relevant time points and the elapsed time between points for each animal. Parameter data were compared between groups using Repeated-Measures Analysis of Variance and a model that included “Time” as a within-subject factor. Treatment, time and treatment × time were compared using Tukey’s All Pairwise Comparison Test at *p* < 0.05. Average daily gain was compared between treatments for the relevant time periods using Analysis of Variance and a model that included “Site”. Means were again compared at *p* < 0.05. Proportions of normal and abnormal disbudding wounds at days 7–8 and 11–12 were compared between groups using Fisher’s Exact Test at *p* < 0.05.

## 3. Results

### 3.1. Study 1—Target Animal Safety Study—Safety of Tri-Solfen^®^ Administration to Calf Disbudding Wounds Including up to 5× the Recommended Dose

#### 3.1.1. Clinical Examinations

Animals remained visibly well throughout the experimental period. There were no recorded serious adverse events, and no mortality of calves during the study period. Group mean values (Table S1 for the key clinical parameters, including rectal temperature, heart rate, respiration rate, water intake, and feed intake) between Group 1 and the treatment groups (Groups 2–4) were similar over time (Table 2) with no evidence of a dose–titration effect. Bodyweights and average daily gain were also similar with no statistically significant differences observed between groups (Table 2).

#### 3.1.2. Haematological and Urine Analysis

Haematological analysis detected a small but statistically significant difference for Red Blood Cells, Haemoglobin and Haemocrit, between Group 1 (placebo) and Group 3 (5× dose group); however, group means remained in the upper normal of the reference range for the duration of the study (Table S2). No other significant differences in haematological parameters (Mean Corpuscular Volume, Mean Corpuscular Haemoglobin, Mean Corpuscular Haemoglobin Concentration, White Blood Cells, Activated Partial Thromboplastin Time, Prothrombin Time and Fibrinogen) were observed.

Group mean values for the key biochemical parameters (Alanine Aminotransferase, Albumin, Alkaline Phosphatase, Aspartate Aminotransferase, Creatinine, Creatine Kinase, Gamma-Glutamyltransferase, Globulin, Lactate Dehydrogenase, Total Protein and Urea) followed similar trends over time with any differences found between groups not statistically significant (Table S3). No divergence between parameters was observed at higher doses.

Urine specimens from animals in each group were analysed for a range of key parameters. A significant difference was noted for detectable blood (based on pseudoperoxidase activity of haemoglobin and myoglobin) between Group 4 (5× dose) and Group 1 (placebo) (*p* < 0.010). No significant differences were observed for any other parameters (Table 2).

#### 3.1.3. Gross Pathology of Animals Following Euthanasia

All animals were euthanised and subjected to necropsy at days 3 and 4 following disbudding and treatment. On gross examination at necropsy, lung consolidation was observed in 10/32 (31.3%) of animals, consistent with the presence of current or historical sub-clinical pneumonia. Of the 10 animals, 3 (30%) were from the placebo group, 2 (20%) were from Group 2, 3 (30%) were from Group 3 and 2 (20%) were from Group 4. No other lesions or abnormalities were observed with any other organ system.

#### 3.1.4. Histopathological Analysis of Tissue Samples, Including Skin

Histopathological analysis of tissue samples was undertaken on all Group 1 (placebo) and Group 4 (5× dose) animals. No evidence of pathology was noted in any organ sample examined.

The microscopic findings from histopathological analysis of skin samples from the wound site are presented in Table 3. Locally extensive or diffuse cutaneous necrosis with haemorrhage, oedema, crusting, mineralisation, inflammatory infiltrates and fibrosis were similar in all calves across Groups 1–4. There was a noticeable reduction in the quantity of surface-localised bacteria, graded using a subjective scale between Groups 2, 3 and 4 (Tri-Solfen^®^ treatment groups) and Group 1, the placebo group.

### 3.2. Study 2: Safety and Efficacy of Tri-Solfen Administration to Calf Disbudding Wounds under Field Conditions

#### 3.2.1. Environmental Conditions

A range of extreme environmental conditions occurred during the study period, including an extended heat wave with daytime temperatures consistently >40 °C. Catastrophic bushfire conditions were declared by the Australian Bureau of Meteorology for the study area. Significant air pollution secondary to bushfire smoke and waves of fly infestation were also experienced during the study observation period.

#### 3.2.2. Clinical Examinations

Detailed individual clinical examinations by a veterinarian were performed on all selected calves on day −2 and day −1 (following sham dehorning) and immediately prior to pain assessments at 22 h post procedure and on days 7–8 and days 11–12. Examination parameters included heart rate, respiration rate, rectal temperature, gastrointestinal activity, gait and locomotion. There was a trend towards higher rectal temperatures in placebo treated animals. Mean rectal temperatures were higher in the placebo treatment group at all time points except days 7–8. The most marked differences occurred on day 1 when group mean rectal temperatures of 39.5 °C and 39.1°C were observed for placebo-treated and Tri-Solfen^®^-treated calves, respectively (*p* = 0.06). No significant differences were observed between treatment groups for heart rate (*p* = 0.53) or respiration rate (*p* = 0.64) (Table 4).

Calves were weighed on day −2, days 11–12, 21–22, and 33–34 and average daily gain was calculated on an individual animal basis over the assessment period. These results are presented in Table 5. There was a trend towards higher average daily gain in Tri-Solfen^®^-treated animals. Average daily gains were higher in the Tri-Solfen^®^ treatment group at all time points, which was particularly notable at days 11–12 (*p* = 0.06).

One animal in the Tri-Solfen^®^ treatment group was found dead in its pen on the morning of day 1. A small amount of consolidated lung tissue was observed post mortem, suggestive of viral pneumonia, with no other abnormalities detected at necroscopy. No other serious adverse events or mortality occurred during the study.

#### 3.2.3. Wound Healing Assessment

Disbudding wounds were visually examined on days 7–8, 11–12, 21–22 and 33–34 and assessed for overall appearance, wound resolution and the presence/absence of infection or other adverse clinical findings. Proportions of normal and abnormal disbudding wounds at each time point are presented in Table 6. Relatively high proportions of wounds were classified as abnormal at days 7–8, (56% and 46% in placebo and Tri-Solfen^®^ treated calves, respectively). While a similar ratio (50%) of abnormal wounds were still present in the placebo group at days 11–12, this number was significantly reduced to 20% (*p* < 0.05) in the Tri-Solfen^®^ treatment group at this time point. The differences between treatment groups disappeared by days 21–22 with wounds continuing to heal normally by day 33–34.

## 4. Discussion

There is an urgent imperative to develop veterinary medicines registered as safe and effective for use to alleviate pain in infant livestock undergoing routine husbandry procedures, including dairy calf disbudding. Applied directly to the disbudding wound, Tri-Solfen^®^ is reported to be effective to mitigate post-operative pain [7,8]. To achieve regulatory approval for such uses, analgesic medicines must meet high standards of proof of safety, requiring detailed proscribed “margin of safety” studies examining the toxic effects of drugs at recommended dose and potential overdose levels. These trials, performed to internationally harmonized standards, are nevertheless invasive and may involve ethical constraints such as the requirement for single product use, where, in non-regulatory trials, multi-modal pain therapy may be used for optimal welfare. The need for such trials is reduced however, wherever sufficient high-quality data are publicly available. Lidocaine and bupivacaine, the local anaesthetics in Tri-Solfen^®^, although widely used in human and veterinary medicine for many decades, are not registered for use to mitigate pain in calves in most jurisdictions. Although their acute systemic neuro- and cardiotoxic effects are well described, unfortunately, at the time of these trials, there was insufficient information available to meet regulatory requirements for proof of safety of use in calves, including lack of information regarding potential wider biochemical, haematological or tissue toxic effects, or impacts when used via topical application to significant open wounds. We therefore have performed regulatory required margin of safety studies with Tri-Solfen^®^ and report the outcome of these to contribute to the public record. It is hoped that these data may reduce the need for such trials for registration of lidocaine or bupivacaine containing products for calves in the future. We separately investigated and reported local anaesthetic pharmacokinetic data in calves following topical application of Tri-Solfen^®^ to the disbudding wound at the recommended dose (2 mL per horn bud) [15]. In the current studies, we report a lack of evidence of significant toxic effects or negative impact on wound healing following Tri-Solfen^®^ use on calf disbudding wounds at up to five times the recommended dose. Conversely a trend towards reduced bacterial load and improved healing was evident in the first 4–14 days following treatment, respectively.

No evidence of significant toxicity was observed in Tri-Solfen^®^-treated animals, with general clinical, biochemical and histopathological data from animals in the placebo and Tri-Solfen^®^ treatment groups similar and remaining within normal parameters despite receiving up to 5× the recommended dose. We did note a minor, but statistically significant difference in the amount of microscopic blood (measured by detection of haemoglobin or myoglobin by dipstick test) detected in the 5× dose group compared to the placebo group animals. This was not associated with an increase in red blood cells in the urine, and there was no evidence of abnormalities detected on histopathological analysis of tissue samples collected from the kidneys, ureters and bladder to suggest any direct toxic effects to any of these tissues. The finding of haemoglobin/myoglobin in urine without an increase in red blood cells could relate to the degree of tissue trauma and/or haemolysis at the wound site. Cauterisation procedures result in muscle tissue and red blood cell damage, releasing myoglobin and haemoglobin, respectively, which may be subsequently reabsorbed and eliminated in the urine. Wound size/depth was not measured as a correlate in this study to assess whether differences may have existed between groups. Histopathological analysis did not note any difference in tissue trauma between groups at the wound site; however, this may not be sensitive to minor differences in red blood cell damage at the time of the procedure or after. There are reports to suggest that high concentrations of lidocaine applied directly to red blood cells may induce haemolysis [51]. It is possible therefore that increased break down of red blood cells contained in crust at the wound site occurred in the 5× treatment group, as compared with the placebo group, contributing to this finding. Intravascular haemolysis is another potential explanation. Although not (to our knowledge) reported in association with bupivacaine administration, rare cases of intravascular haemolysis have been reported in association with lidocaine administration [52]. Typically, however, this is only in the setting of acute toxicity with other factors predisposing to oxidative stress and methaemoglobinaemia [52]. On the other hand, other reports have documented that lidocaine provides a protective effect against intravascular haemolysis [53]. There was a low grade but statistically significant reduction in red blood cells and haemoglobin in blood in animals in the 5× dose group (Group 4) compared to the placebo (Group 1) group; however, haemoglobin and red blood cell values in both groups remained well within the normal range for calves, and mean corpuscular volume and haemoglobin concentration were also not significantly different between groups. This suggests that, if intravascular haemolysis was present in the 5× overdose group, it was of a very low grade and insufficient to have a significant negative impact on animal health.

We also found no evidence of negative impacts on animal health following Tri-Solfen^®^ treatment under field conditions in Study 2, despite the unanticipated extreme environmental conditions that occurred over the course of the study. One animal in the Tri-Solfen^®^-treated group was found to have died 24 h following treatment; however, this was considered unlikely to be related to treatment as there were no clinical signs of toxicity at earlier time points such as in the early minutes and up to 4–6 h post-administration when local anaesthetic concentrations were likely to have peaked [15], such as to suggest acute local anaesthetic systemic toxicity or a rare hypersensitivity reaction [54,55]. Furthermore, the only abnormality detected at necroscopy was pneumonia, and calf survival with pneumonia may have been compromised by the extreme environmental conditions.

These data are thus considered to be consistent with previous data reported in piglets, cattle and sheep, showing an absence of local or systemic toxic effects of Tri-Solfen^®^ treatment at recommended doses during and/or after surgical husbandry procedures [9,28,38,41,42]. They are also consistent with previous studies, limited in number, identifying an absence of local anaesthetic-related toxic effects on haematological, biochemical and/or tissues parameters, when applied at doses that do not induce CNS or CVS toxicity [15,56,57,58,59]. Collectively, these data are thus considered to support the conclusion that the local anaesthetics, as formulated in Tri-Solfen^®^, are minimally absorbed across the thermocautery disbudding wound in calves, and do not generate acute toxic effects, despite administration to up to 5× the maximum recommended dose.

In terms of local application site toxicity, our results are also consistent with previous published data suggesting little negative impact of lidocaine and bupivacaine topical application to open wounds, when used in concentrations commensurate with those present in Tri-Solfen^®^ [21,22,23,24,25,26,27,28,29]. In our target animal safety trial (Study 1), despite the application of doses of Tri-Solfen^®^ significantly greater than the recommended dose, no differences in wound healing were observed as compared with placebo-treated animals over the duration of the trial. These gross observations were confirmed by histopathological analysis of wound biopsies with extensive tissue necrosis observed in all animals, regardless of treatment, indicative of damage caused by the disbudding procedure itself and no evidence of any additional adverse effect triggered by the application of local anaesthetics such as secondary to cytotoxicity. This lack of evidence for any deleterious cytotoxic activity in Tri-Solfen^®^-treated animals was confirmed in our field trials with disbudding wounds in placebo and all treatment groups showing similar healing at day 33–34 following treatment. These results are also consistent with the recent work of Stilwell and Laven [40], which failed to find any deleterious effect on wound healing in Tri-Solfen^®^-treated animals one week after disbudding and treatment as compared with animals treated with topical tetracycline.

While no deleterious effects on wound healing were observed, by contrast, there was evidence of improved wound healing over the first 1–2 weeks following treatment in Tri-Solfen^®^-treated animals. Placebo-treated calves had higher numbers of abnormal wounds, attributed to wound infection by veterinary inspection, on day 11–12 in the field trial (Study 2). This was associated with higher rectal temperatures and reduced average daily gain in placebo as compared with Tri-Solfen^®^-treated calves over the same period. This is suggestive of increased infection rates and greater “set-back” in placebo-treated animals over the first 1–2 weeks following the procedure. “Set-back” involving reduced average daily gain is common in animal post-surgical husbandry procedures and is attributed to increased catabolic rate and/or reduced feed intake secondary to pain and the surgical stress response to tissue trauma [60]. This may be exacerbated by wound infection. This is typically followed by a “catch-up” phase as the wounds heal and the surgical stress response abates [61]. Results from our field trial suggested Tri-Solfen^®^ treatment contributed to improved early wound healing and reduced set-back in the first 1–2 weeks following the procedure. These results are consistent with those reported by others. Lomax et al. similarly reported faster rates of wound healing in Tri-Solfen^®^- versus placebo-treated lambs in the first 14 days following surgical husbandry procedures for flystrike prevention [28]. Cuttance et al. [7] reported a tendency (*p* = 0.09) towards improved average daily gain in calves disbudded with pre-operative sedation, and post-procedural Tri-Solfen^®^ treatment, as compared to those disbudded without treatment. Van de Saag et al. [62] reported improved weight gain in calves castrated and disbudded when treated with meloxicam and Tri-Solfen^®^, as compared with untreated animals. It is not known whether such effects may relate to reductions in pain, reduction in inflammation and the surgical stress response, and/or reduction in bacterial colonisation and wound infection rates, or a combination of the three.

Evidence for the potential beneficial antimicrobial effect of Tri-Solfen^®^ treatment and a potential explanation for the improved early wound healing we observed in our field trial can be seen in our observations of microbial colonisation of disbudding wounds following histopathological analysis. Reduced microbial colonisation of the wounds was evident in Tri-Solfen^®^ as compared with placebo-treated animals from samples collected at necroscopy (i.e., 3–4 days following treatment). Similar effects (reduced pus discharge and bacterial colony counts) have been reported following use of cetrimide containing antiseptics on surgical wounds in dogs [63], suggesting that it is likely that this effect is due to the presence of the antiseptic, cetrimide, in the topical anaesthetic formulation. Local anaesthetics also possess some antibacterial activity [32,64] that may have contributed to a reduction in the microbial load in the disbudding wound, and, hypothetically, the reduced risk of abnormal wounds we observed at day 11/12 in Study 2. More detailed studies to characterize the wound healing efficacy and antimicrobial activity of Tri-Solfen^®^ treatment following disbudding are warranted, particularly if it can be shown that Tri-Solfen^®^ treatment can reduce the incidence of wound infections similarly or with greater efficacy than the prophylactic use of topical antimicrobial sprays. This has potential beneficial effects to reduce the need for antibiotic use both for prophylaxis and/or to treat wound infections, the latter antibiotic treatments contributing to the emergence of antimicrobial resistance [65].

## 5. Conclusions

The results of these safety studies reveal that the topical application of lidocaine and bupivacaine with adrenaline and cetrimide, as present in Tri-Solfen^®^, to disbudding wounds in calves presents a minimal risk of acute local or systemic toxicity even at doses significantly higher than the recommended dosage. At recommended and overdose levels, no evidence could be found for an adverse effect on wound healing. Conversely, Tri-Solfen^®^ treatment may have beneficial impacts on wound healing, possibly secondary to lower bacterial colonisation and the incidence of wound infections.

## Figures and Tables

**Table 1:** Treatment regime for animals in Study 1.

Group	Animals (*n*)	Treatment	Dose level	Dosing Regime
1	8	Placebo	-	2 mL sterile saline applied on Days 0, 1 and 2 (2 mL per day total)
2	8	Tri-Solfen^®^	1×	2 mL Tri-Solfen^®^ applied daily on Days 0, 1 and 2 (2 mL per day total)
3	8	Tri-Solfen^®^	3×	2 mL Tri-Solfen^®^ applied three times at 1 h intervals on Days 0, 1 and 2 (6 mL per day total)
4	8	Tri-Solfen^®^	5×	2 mL Tri-Solfen^®^ applied five times at 1 h intervals on Days 0, 1 and 2 (10 mL per day total)

**Table 2:** Statistical analysis of key safety parameters and observed *p*-Values for treatment (and time as appropriate). Statistically significant results are indicated in bold.

Parameter	*p*-Value (Treatment)	*p*-Value (Time)
Bodyweight	0.117	<0.001
Rectal Temperature	0.727	0.482
Heart Rate	0.443	<0.001
Respiration Rate	0.601	<0.001
Average Daily Gain	0.126	-
Water Intake	0.998	<0.001
Feed Intake	0.026	<0.001
Red Blood Cells	**0.003**	0.081
Haemoglobin	**0.004**	0.112
Haematocrit	**0.003**	0.003
White Blood Cells	0.320	0.342
Mean Corpuscular Volume	0.596	<0.001
Mean Corpuscular Haemoglobin	0.518	0.635
Mean Corpuscular Haemoglobin Concentration	0.430	0.002
Activated Partial Thromboplastin Time	0.056	0.500
Prothrombin Time	0.056	0.166
Fibrinogen	0.168	0.000
Alanine aminotransferase	0.151	0.138
Albumin	0.431	0.000
Alkaline phosphatase	0.595	<0.001
Aspartate aminotransferase	0.943	<0.001
Creatinine	0.225	<0.001
Log 2 Creatine Kinase	0.829	0.124
Gamma-glutamyltransferase	0.468	0.006
Globulin	0.048	0.000
Lactate Dehydrogenase	0.230	0.001
Total protein	0.596	0.169
Urea	0.631	0.247
Colour	0.886	-
Turbidity	1.000	-
Blood	**0.010**	-
Protein	0.832	-
Bilirubin	1	-
Red Blood Cells	0.895	-
White Blood Cells	0.126	-
Unidentified Crystalline Structures	0.886	-
Bilirubin Crystals	0.886	-
Amorphous Urate Crystals	0.587	-
Struvite Crystals	0.893	-
Epithelial Cells	0.587	-
Amorphous Debris	0.073	-
pH	0.060	-
Specific Gravity	0.569	-

**Table 3:** Incidence of pathological conditions and/or the presence of multi-focal surface bacterial colonies in skin samples collected from animals in Study 1.

Finding ^a^	Group 1 (*n* = 8)	Group 2 (*n* = 8)	Group 3 (*n* = 8)	Group 4 (*n* = 8)
Locally extensive or diffuse epidermal coagulative necrosis with haemorrhage, oedema, neutrophilic infiltrates, serum crusting, occasional mineralisation	-	-	-	-
0	0	0	0	0
1	0	0	0	0
2	0	0	0	0
3	0	0	0	0
4	8	8	8	8
Surface bacterial colonies, multifocal	-	-	-	-
0	0	0	0	1
1	1	4	5	7
2	2	0	1	0
3	1	3	0	0
4	4	1	2	0
Dermal perivascular lymphoplasmacytic, neutrophilic and eosinophilic infiltrates, multifocal	-	-	-	-
0	0	0	0	0
1	0	0	0	0
2	5	6	7	4
3	3	2	1	4
4	0	0	0	0
Dermal fibrosis	-	-	-	-
0	0	0	0	0
1	2	1	0	4
2	2	5	6	1
3	4	2	2	3
4	0	0	0	0

^a^ Grading scale; 0 = normal, 1 = minimal, 2 = mild, 3 = moderate, 4 = marked.

**Table 4:** Mean clinical data (rectal temperature, heart rate, respiratory rate) for Placebo and Tri-Solfen^®^-treated calves over time in Study 2.

Group/Treatment	Rectal Temperature (°C)	Heart Rate (beats/min)	Respiratory Rate (breaths/min)
Day −2			
Placebo	38.8 ± 0.49	128.9 ± 19.3	44.0 ± 8.1
Tri-Solfen^®^	38.7 ± 0.53	128.4 ± 22.7	47.0 ± 10.6
Day −1			
Placebo	38.8 ± 0.30	129.3 ± 20.0	41.5 ± 9.2
Tri-Solfen^®^	38.7 ± 0.36	128.5 ± 26.2	40.4 ± 11.0
Day 1	-	-	-
Placebo	39.5 ± 0.50	125.1 ± 14.6	44.5 ± 10.2
Tri-Solfen^®^	39.1 ± 0.46	131.0 ± 20.7	43.7 ± 9.7
Day 7–8			
Placebo	39.9 ± 0.52	117.1 ± 21.3	51.7 ± 19.5
Tri-Solfen^®^	39.9 ± 0.55	121.1 ± 27.2	50.7 ± 17.8
Day 11–12			
Placebo	39.3 ± 0.51	123.8 ± 20.7	44.2 ± 10.9
Tri-Solfen^®^	39.1 ± 0.55	124.7 ± 19.4	43.1 ± 10.9

**Table 5:** Average daily gain (kg/day) in calves from each treatment group over time for Study 2.

Group/Treatment	Average Daily Gain (Day −2 to Day 11–12) kg/day	Average Daily Gain (Day −2 to Day 21–22) kg/day	Average Daily Gain (Day −2 to Day 33–34) kg/day
Placebo	0.65	0.81	0.83
Tri-Solfen^®^	0.85	0.89	0.88
Treatment effect (Average Daily Gain)	0.20	0.08	0.05
Treatment effect (%)	31	10	6

**Table 6:** Proportions of normal versus abnormal disbudding wounds in animals between treatment groups and over time for Study 2.

Timepoint	Treatment Group	Abnormal (%)	Normal (%)
Days 7–8	Placebo (*n* = 36)	20 (55.6)	16 (44.4)
	Tri-Solfen^®^ (*n* = 35)	16 (45.7)	19 (54.3)
Days 11–12	Placebo (*n* = 34)	17 (50.0)	17 (50.0)
	Tri-Solfen^®^ (*n* = 35)	7 (20.0)	28 (80.0)
Days 21–22	Placebo (*n* = 36)	3 (8.3)	33 (91.7)
	Tri-Solfen^®^ (*n* = 36)	3 (8.3)	33 (91.7)
Days 33–34	Placebo (*n* = 36)	0 (0.0)	36 (100)
	Tri-Solfen^®^ (*n* = 36)	0 (0.0)	36 (100)

## Data Availability

The data presented in this study are available at https://www.mdpi.com/2076-2615/11/3/869/s1.

## Supplementary Materials

The following are available online at https://www.mdpi.com/2076-2615/11/3/869/s1, Table S1: Group mean key clinical parameters over time for animals in Experiment 1, Table S2: Group mean key haematological parameters over time for animals in Experiment 1, Table S3: Group mean key biochemical parameters over time for animals in Experiment 1.
