# Supplementary material for: Topical Application of Lidocaine and Bupivacaine to Disbudding Wounds in Dairy Calves: Safety, Toxicology and Wound Healing
Source: Animals (Basel). 2021 Mar 18;11(3):869. doi: 10.3390/ani11030869 (PMC8003238; doi:10.3390/ani11030869)
Supplement: Supplementary file 1 [file animals-11-00869-s001.zip › Revised Table S1 - Mean clinical parameters.docx]

**Table S1.** Group mean key clinical parameters over time for animals in Study 1.

| **Parameter / Treatment Group** | **Units** | **1.Placebo** | **2. TRI-SOLFEN .1X** | **3. TRI-SOLFEN. 3X** | **4.TRI-SOLFEN.5X** |
| --- | --- | --- | --- | --- | --- |
|  |  | **Day -4** | | | |
| Bodyweight | kg | 49.4 | 47.9 | 50.2 | 47.7 |
| Temperature | °C | 38.16 | 38.50 | 38.24 | 38.33 |
| Heart Rate | beats/minute | 108.5 | 112.0 | 102.0 | 112.3 |
| Respiration Rate | breaths/minute | 47.0 | 51.0 | 48.0 | 47.0 |
|  |  | **Day 0** | | | |
| Bodyweight | kg | 0 | 0 | 0 | 0 |
| Temperature | °C | 38.76 | 38.58 | 38.73 | 38.71 |
| Heart Rate | beats/minute | 121.5 | 122.5 | 121.8 | 115.0 |
| Respiration Rate | breaths/minute | 50.5 | 50.0 | 48.5 | 48.0 |
|  |  | **Day 3/4** | | | |
| Bodyweight | kg | 53.0 | 57.0 | 54.6 | 53.1 |
| Temperature | °C | 38.66 | 38.59 | 38.63 | 38.70 |
| Heart Rate | beats/minute | 107.0 | 115.5 | 92.5 | 98.5 |
| Respiration Rate | breaths/minute | 28.8 | 30.6 | 26.1 | 28.6 |
