# Supplementary material for: Topical Application of Lidocaine and Bupivacaine to Disbudding Wounds in Dairy Calves: Safety, Toxicology and Wound Healing
Source: Animals (Basel). 2021 Mar 18;11(3):869. doi: 10.3390/ani11030869 (PMC8003238; doi:10.3390/ani11030869)
Supplement: Supplementary file 1 [file animals-11-00869-s001.zip › Revised Table S2 - Group mean haematological parameters over time.docx]

**Table S2.** Group mean key haematological parameters over time for animals in Study 1.

| **Parameter** | **Units** | **Treatment Group / Reference Range** | **1.Placebo** | **2.TRI-SOLFEN.1X** | **3.TRI-SOLFEN.3X** | **4.TRI-SOLFEN.5X** |
| --- | --- | --- | --- | --- | --- | --- |
|  |  |  | **Day -4** | | | |
| Red Blood Cells | x 10^12^/L | 5.11 - 11.06 | 11.29 | 11.43 | 11.23 | 11.28 |
| Haemoglobin | g/L | 68.5 - 145.6 | 123.3 | 127.8 | 128.4 | 127.8 |
| Haematocrit | L/L | 0.18 - 0.46 | 0.396 | 0.405 | 0.410 | 0.409 |
| White Blood Cells | x 10^9^/L | 4.30 - 14.80 | 10.43 | 10.55 | 10.93 | 12.08 |
| Mean Corpuscular Volume | fL | 33.2 - 46.6 | 35.0 | 35.5 | 36.6 | 36.4 |
| Mean Corpuscular Haemoglobin | pg | 10.99 - 14.62 | 10.8 | 11.1 | 11.6 | 11.4 |
| Mean Corpuscular Haemoglobin Concentration | g/L | 302.6 - 341.2 | 311.4 | 315.8 | 314.5 | 313.0 |
| Activated Partial Thromboplastin Time | seconds | 25.3 - 44.5 | 62.3 | 69.9 | 74.1 | 74.9 |
| Prothrombin Time | seconds | 20.1 - 30.1 | 22.3 | 22.2 | 33.6 | 24.5 |
| Fibrinogen | g/L | 2 - 7 | 4.63 | 3.63 | 5.38 | 5.00 |
|  |  |  | **Day 0** | | | |
| Red Blood Cells | x 10^12^/L | 5.11 - 11.06 | 10.86 | 11.16 | 11.00 | 10.28 |
| Haemoglobin | g/L | 68.5 - 145.6 | 117.6 | 123.6 | 125.5 | 116.8 |
| Haematocrit | L/L | 0.18 - 0.46 | 0.380 | 0.398 | 0.401 | 0.375 |
| White Blood Cells | x 10^9^/L | 4.30 - 14.80 | 9.59 | 10.90 | 11.35 | 10.93 |
| Mean Corpuscular Volume | fL | 33.2 - 46.6 | 35.0 | 35.4 | 36.6 | 36.3 |
| Mean Corpuscular Haemoglobin | pg | 10.99 - 14.62 | 10.9 | 11.1 | 11.4 | 11.4 |
| Mean Corpuscular Haemoglobin Concentration | g/L | 302.6 - 341.2 | 309.1 | 312.8 | 313.0 | 313.5 |
| Activated Partial Thromboplastin Time | seconds | 25.3 - 44.5 | 56.3 | 53.6 | 58.3 | 58.1 |
| Prothrombin Time | seconds | 20.1 - 30.1 | 35.8 | 37.2 | 34.5 | 24.8 |
| Fibrinogen | g/L | 2 - 7 | 4.50 | 4.25 | 4.50 | 5.13 |
|  |  |  | **Day 3/4** | | | |
| Red Blood Cells | x 10^12^/L | 5.11 - 11.06 | 10.80 | 11.03 | 10.91 | 9.99 |
| Haemoglobin | g/L | 68.5 - 145.6 | 117.3 | 122.0 | 124.3 | 113.4 |
| Haematocrit | L/L | 0.18 - 0.46 | 0.375 | 0.388 | 0.393 | 0.359 |
| White Blood Cells | x 10^9^/L | 4.30 - 14.80 | 10.11 | 10.01 | 12.03 | 12.34 |
| Mean Corpuscular Volume | fL | 33.2 - 46.6 | 34.6 | 35.1 | 36.0 | 35.8 |
| Mean Corpuscular Haemoglobin | pg | 10.99 - 14.62 | 10.8 | 11.1 | 11.5 | 11.5 |
| Mean Corpuscular Haemoglobin Concentration | g/L | 302.6 - 341.2 | 312.8 | 315.8 | 316.9 | 316.0 |
| Activated Partial Thromboplastin Time | seconds | 25.3 - 44.5 | 56.8 | 54.2 | 57.1 | 62.4 |
| Prothrombin Time | seconds | 20.1 - 30.1 | 31.9 | 27.2 | 32.0 | 28.8 |
| Fibrinogen | g/L | 2 - 7 | 6.00 | 5.88 | 5.63 | 7.50 |
