# Supplementary material for: Topical Application of Lidocaine and Bupivacaine to Disbudding Wounds in Dairy Calves: Safety, Toxicology and Wound Healing
Source: Animals (Basel). 2021 Mar 18;11(3):869. doi: 10.3390/ani11030869 (PMC8003238; doi:10.3390/ani11030869)
Supplement: Supplementary file 1 [file animals-11-00869-s001.zip › Revised Table S3 - Group mean key biochemical parameters over time in animals from Experiment 1.docx]

**Table S3.** Group mean key biochemical parameters over time for animals in Study 1.

| **Parameter** | **Units** | **Treatment Group / Reference Range** | **1.Placebo** | **2.TRI-SOLFEN.1X** | **3.TRI-SOLFEN.3X** | **4.TRI-SOLFEN.5X** |
| --- | --- | --- | --- | --- | --- | --- |
|  |  |  | **Day -4** | | | |
| Alanine aminotransferase | IU/L | 0 - 83 | 8.38 | 7.63 | 8.88 | 7.75 |
| Albumin | g/L | 26 - 37 | 33.0 | 32.5 | 32.4 | 31.9 |
| Alkaline phosphatase | IU/L | 0 - 318 | 258 | 282 | 241 | 280 |
| Aspartate aminotransferase | IU/L | 12 - 56 | 49.6 | 47.5 | 57.0 | 47.5 |
| Creatinine | µmol/L | 61 - 127 | 64.1 | 71.3 | 65.3 | 62.9 |
| Creatine Kinase | IU/L | 0 - 250 | 169 | 185 | 530 | 166 |
| Gamma-glutamyltransferase | IU/L | 0 - 68 | 50.0 | 37.5 | 44.3 | 31.4 |
| Globulin | g/L |  | 25.9 | 25.8 | 26.0 | 27.3 |
| Lactate Dehydrogenase | u/L | 0 - 3258 | 789 | 731 | 824 | 800 |
| Total protein | g/L | 45 - 61 | 58.8 | 58.1 | 58.3 | 58.9 |
| Urea | mmol/L | 1.6 - 6.5 | 3.30 | 2.94 | 2.98 | 2.98 |
|  |  |  | **Day 0** | | | |
| Alanine aminotransferase | IU/L | 0 - 83 | 9.13 | 8.88 | 9.25 | 8.88 |
| Albumin | g/L | 26 - 37 | 32.5 | 32.8 | 32.1 | 31.9 |
| Alkaline phosphatase | IU/L | 0 - 318 | 263 | 289 | 240 | 292 |
| Aspartate aminotransferase | IU/L | 12 - 56 | 55.6 | 55.5 | 63.4 | 55.1 |
| Creatinine | µmol/L | 61 - 127 | 66.5 | 74.4 | 68.3 | 62.4 |
| Creatine Kinase | IU/L | 0 - 250 | 223 | 189 | 262 | 256 |
| Gamma-glutamyltransferase | IU/L | 0 - 68 | 37.3 | 31.0 | 37.1 | 26.6 |
| Globulin | g/L |  | 25.4 | 25.3 | 27.0 | 26.8 |
| Lactate Dehydrogenase | u/L | 0 - 3258 | 832 | 820 | 916 | 897 |
| Total protein | g/L | 45 - 61 | 57.8 | 58.0 | 59.0 | 58.6 |
| Urea | mmol/L | 1.6 - 6.5 | 3.35 | 3.28 | 3.24 | 3.08 |
|  |  |  | **Day 3/4** | | | |
| Alanine aminotransferase | IU/L | 0 - 83 | 9.00 | 8.00 | 8.63 | 8.75 |
| Albumin | g/L | 26 - 37 | 33.6 | 33.9 | 33.4 | 32.9 |
| Alkaline phosphatase | IU/L | 0 - 318 | 214 | 256 | 198 | 225 |
| Aspartate aminotransferase | IU/L | 12 - 56 | 50.9 | 50.5 | 56.3 | 49.4 |
| Creatinine | µmol/L | 61 - 127 | 58.5 | 65.3 | 59.8 | 55.5 |
| Creatine Kinase | IU/L | 0 - 250 | 172 | 195 | 248 | 202 |
| Gamma-glutamyltransferase | IU/L | 0 - 68 | 30.6 | 29.6 | 30.0 | 25.4 |
| Globulin | g/L |  | 24.8 | 23.6 | 24.6 | 25.0 |
| Lactate Dehydrogenase | u/L | 0 - 3258 | 796 | 788 | 881 | 831 |
| Total protein | g/L | 45 - 61 | 58.4 | 57.3 | 58.1 | 57.8 |
| Urea | mmol/L | 1.6 - 6.5 | 3.28 | 3.25 | 3.13 | 2.99 |
